# Intramuscular Cavernous Hemangioma of the Vastus Medialis Muscle in a Young High School Athlete: A Rare Cause of Chronic Anterior Knee Pain

**DOI:** 10.7759/cureus.111841

**Published:** 2026-06-30

**Authors:** Kannan Murthy, Youssef Messiha, Vansh Rana, Ussef Najib

**Affiliations:** 1 Orthopedics, Kern Medical Center, Bakersfield, USA; 2 Orthopedics, University of California, Los Angeles (UCLA)-Kern Medical Center, Bakersfield, USA; 3 Orthopedic Surgery, University of California, Los Angeles (UCLA)-Kern Medical Center, Bakersfield, USA

**Keywords:** adolescent athlete, intramuscular hemangioma, knee pain, vascular tumor, vastus medialis

## Abstract

Intramuscular hemangiomas are rare benign vascular tumors of skeletal muscle that are frequently misdiagnosed due to their deep location and vague presentation. These lesions may present similarly to common orthopedic pathology.

We report a 17-year-old male high school athlete with chronic, progressively worsening anteromedial knee pain following prior trauma, associated with progressive swelling. Symptoms persisted despite conservative management, limiting sports participation without impairment in ambulation.

The outside radiology report of the patient showed a cystic-appearing lesion in the anteromedial aspect of the knee, initially interpreted as consistent with a ganglion cyst, and less likely a malignancy. Given the chronicity of symptoms and functional limitation, surgical excision was indicated. Surgical pathology report confirmed an intramuscular hemangioma.

Postoperatively, the patient experienced improvement in pain with no signs of early recurrence at two-week follow-up.

Intramuscular hemangioma should be considered in adolescents with chronic periarticular pain or deep soft-tissue lesions, particularly when symptoms significantly limit activity, chronicity is involved, and imaging suggests an intramuscular vascular process. Surgical excision can be diagnostic and potentially curative when complete resection is achieved, and awareness of this pathology may reduce delays in diagnosis and unnecessary interventions.

## Introduction

An intramuscular hemangioma (IMH) is a rare benign vascular tumor that arises within skeletal muscle and accounts for approximately 0.8% of all hemangiomas; hemangiomas overall represent roughly 7% of benign soft-tissue tumors [1,2]. Allen and Enzinger first established the histopathologic framework for these lesions in their landmark review of 89 cases at the Armed Forces Institute of Pathology, subclassifying them by predominant vessel caliber into small-vessel (capillary), large-vessel (cavernous), and mixed types [2]. IMH is characterized by the proliferation of benign vascular channels that infiltrate muscle fibers. It has historically been referred to by several terms, including intramuscular angioma and intramuscular capillary-type hemangioma [3]. According to the current World Health Organization classification of soft-tissue and bone tumors, IMH is considered a benign vascular tumor distinct from vascular malformations [4,5]. The International Society for the Study of Vascular Anomalies (ISSVA) classification likewise distinguishes true vascular tumors such as IMH from low-flow and high-flow vascular malformations, a clinically relevant distinction that informs imaging interpretation and treatment selection [6]. Although modern ISSVA terminology separates vascular tumors from vascular malformations, the entrapment of skeletal muscle fibers within the vascular stroma seen here is a defining feature of IMH and distinguishes it from a venous malformation occupying the same plane.

IMH most commonly affects children and young adults, with the majority of cases diagnosed before age 30 [3]. In Beham and Fletcher’s clinicopathologic review of 74 cases, the mean age at presentation was 17.6 years, 85% of patients presented before age 30, and the lower limb accounted for approximately 32% of lesions, demographics that closely match the present case [7]. IMH can occur in any muscle group and shows a predilection for the extremities, particularly the thigh and calf. Its occurrence in periarticular structures such as the knee is uncommon, and nonspecific symptoms make the diagnosis particularly difficult in this location. Clinical presentation may include activity-related pain, swelling, or restricted range of motion. When the lesion occurs near a major joint, such as the knee, a region where symptoms can closely mimic common orthopedic pathology, early recognition is crucial to avoid unnecessary interventions and to preserve function. Imaging, particularly magnetic resonance imaging (MRI), is essential for identifying an intramuscular lesion [8]. This report describes a case of an intramuscular cavernous hemangioma of the knee in a young male, highlighting the diagnostic challenges, preoperative presentation, and surgical considerations unique to lesions in this anatomic region.

## Case presentation

The patient was a 17-year-old male who presented with several months of chronic right-knee pain that interfered with athletic activity. He localized the pain to the anteromedial knee with focal tenderness and recalled a remote knee injury approximately two years prior. He denied constitutional symptoms such as fever, chills, and unintentional weight loss, and he denied interval enlargement of the palpable lesion. He remained able to ambulate without instability but reported pain-limited activity.

His past medical history was unremarkable, and he had no prior orthopedic or knee procedures. His only current medication was naproxen.

On examination, the patient was alert, oriented, and in no acute distress. Cardiopulmonary examination was unremarkable. Focused musculoskeletal examination of the right knee demonstrated intact overlying skin and focal tenderness at the anteromedial knee near the vastus medialis insertion on the patella. A small, palpable, cystic-appearing mass was appreciated in this region and appeared to transilluminate. Active and passive range of motion was full from 0° to 130° (extension to flexion). Ligamentous examination was stable to varus and valgus stress, with negative Lachman and posterior drawer maneuvers. The contralateral knee examination was within normal limits. Distal neurovascular status was intact with preserved motor and sensory function and capillary refill of less than two seconds. Vital signs were within normal limits.

Plain radiographs of the right knee showed no acute fracture or dislocation. MRI of the right knee without intravenous contrast (November 2024, Kern Radiology Medical Group) demonstrated a complex, well-circumscribed, predominantly cystic lesion within the distal vastus medialis proximal to the myotendinous junction, measuring up to 21.0 mm (Figure 1). The adjacent musculature was unremarkable without evidence of invasion or edema. Radiologic differential included IMH, ganglion cyst, vascular malformation, cystic hygroma, and post-traumatic hematoma, with malignancy felt to be less likely. The lesion was characterized on non-contrast sequences only; specific T1/T2 signal features, internal septations, flow voids, and phleboliths were not detailed, and ultrasound to assess vascularity was recommended. In retrospect, contrast-enhanced MRI and the recommended ultrasound could have helped distinguish a vascular lesion from a simple ganglion cyst preoperatively.

**Figure 1:**
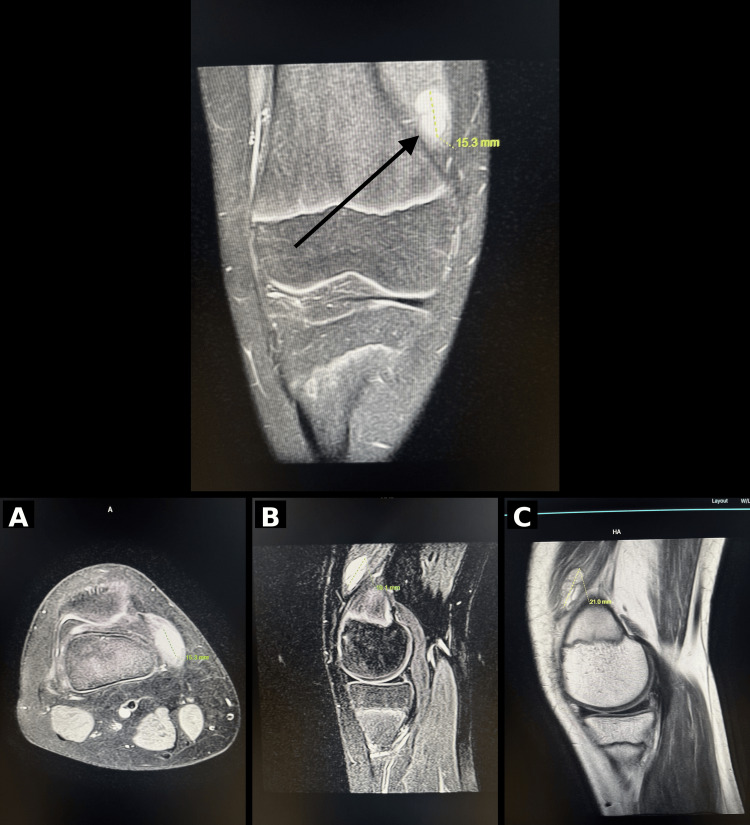
Preoperative MRI of the right knee demonstrating an intramuscular lesion within the distal vastus medialis. Preoperative non-contrast MRI of the right knee. (A) Axial T2 fat-suppressed image: well-circumscribed intramuscular lesion in the distal vastus medialis (approx. 15.3 mm). (B) Coronal T2 fat-suppressed image: maximal dimension 19.1 mm. (C) Sagittal T2 image: approx. 21.0 mm.

The differential diagnosis included ganglion cyst, chondromalacia patellae, parameniscal cyst, bursitis, lipoma, IMH, and soft-tissue neoplasm. A ganglion cyst was favored preoperatively because the lesion was cystic-appearing, transilluminated on examination, and demonstrated benign, non-aggressive features on non-contrast MRI. Importantly, the study was performed without intravenous contrast, which limits characterization of vascular lesions: on non-contrast sequences, IMHs may appear predominantly cystic, and features such as phleboliths, internal septations, or flow voids may be absent or inconspicuous. The interpreting radiologist accordingly recommended ultrasound to assess vascularity and noted that tissue diagnosis might ultimately be necessary. The absence of these vascular markers on a non-contrast study, therefore, did not exclude a hemangioma; rather, the vascular nature of the lesion was recognized only on histopathology.

At the initial orthopedic visit, nonoperative and operative management options were discussed. Nonoperative management consisted of symptomatic treatment, including rest, ice, compression, and elevation (RICE) therapy and over-the-counter (OTC) medications. The patient elected against continued nonoperative management because his pain had been persistent for several months without significant improvement. Biopsy with referral to orthopedic oncology was discussed; however, the patient preferred to pursue management within his hometown. Based on the clinical presentation and imaging findings, the working diagnosis favored a benign cystic lesion (ganglion cyst) that appeared to transilluminate. Malignancy was considered less likely at that point. An IMH was not suspected preoperatively. Excisional biopsy was pursued based on the convergence of three factors: (1) persistent, activity-limiting anterior knee pain refractory to conservative management; (2) diagnostic uncertainty, as the non-contrast MRI could not definitively distinguish between the differentials; and (3) the interpreting radiologist's recommendation that tissue diagnosis may ultimately be necessary. Given the lesion's small size, well-circumscribed margins, absence of invasion or surrounding edema, and benign imaging characteristics, marginal excision was considered a reasonable approach that would simultaneously address the patient's symptoms and provide a definitive histologic diagnosis.

The patient underwent right-knee mass excision with biopsy under general endotracheal anesthesia. The mass was localized with ultrasound guidance. Intraoperatively, a 4 cm longitudinal incision was made over the vastus medialis oblique (VMO). Dissection through the superficial VMO revealed the mass deep to the superficial muscle layer. The mass was resected using electrocautery with a margin of normal-appearing muscle circumferentially (marginal excision) and was sent to pathology (Figure 2).

**Figure 2:**
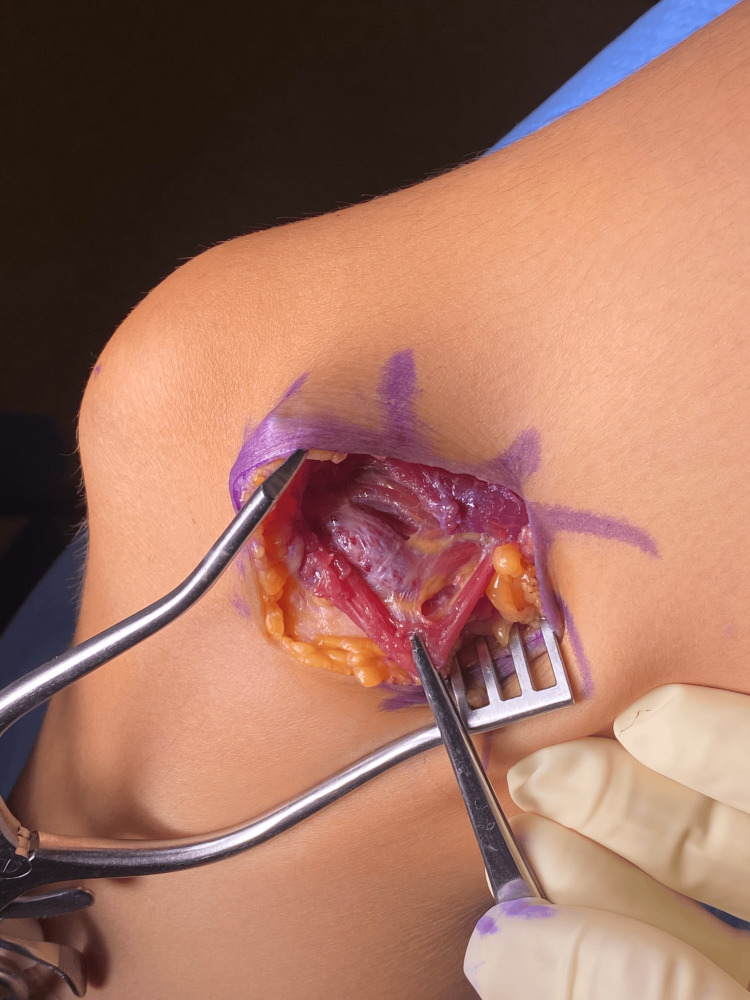
Intraoperative exposure of the intramuscular lesion within the vastus medialis oblique. Intraoperative photograph obtained through a 4 cm longitudinal anteromedial incision over the vastus medialis oblique (VMO). After dissection through the superficial VMO, the mass was identified deep to the superficial muscle layer and is shown grasped with forceps prior to circumferential excision with a cuff of normal-appearing muscle. Final surgical pathology confirmed an intramuscular hemangioma.

A tourniquet was used, inflated to 250 mmHg and maintained for 30 minutes. The wound was irrigated, 1 g of intravenous vancomycin prophylaxis was administered prior to closure, and the wound was closed in layers and dressed sterilely with no intraoperative complications.

Postoperatively, the patient was instructed to begin daily knee range-of-motion exercises and to weight-bear as tolerated with light activity. Venous thromboembolism prophylaxis consisted of sequential compression devices (SCDs) intraoperatively, low-dose aspirin (81 mg daily), and early postoperative ambulation. At the two-week follow-up, pain was well controlled. Examination demonstrated mild effusion without erythema, warmth, or other signs of infection. Final pathology with hematoxylin and eosin staining revealed histologic features classic for intramuscular cavernous hemangioma, rendering immunohistochemical studies unnecessary for diagnostic confirmation (Figure 3). Additional follow-up was planned; however, the patient was lost to follow-up beyond the two-week postoperative visit, and unfortunately, return-to-sport status was not assessed.

**Figure 3:**
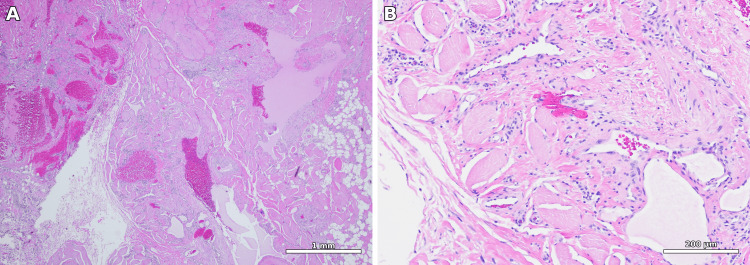
Histopathology of the resected vastus medialis intramuscular hemangioma (H&E). Hematoxylin and eosin (H&E)-stained sections of the resected vastus medialis intramuscular hemangioma. (A) Low-power photomicrograph (2x objective) demonstrating dilated, blood-filled vascular channels of variable caliber dissecting between residual skeletal muscle bundles, with focal adipose admixture, consistent with the cavernous subtype of intramuscular hemangioma. (B) Higher-power photomicrograph (10x objective) highlighting entrapment of skeletal muscle fibers within the vascular stroma – a defining histologic feature distinguishing intramuscular hemangioma from a vascular malformation occupying the same anatomic plane. Formal microscopic margin status was not specifically reported; the lesion was grossly excised with a circumferential cuff of normal muscle. Scale bars: 1 mm (A); 200 µm (B).

## Discussion

IMHs are rare benign vascular tumors of mesodermal origin that most commonly arise in skeletal muscle and frequently present a diagnostic challenge because of their deep location and nonspecific symptoms. Although the exact etiology remains unclear, proposed mechanisms include congenital vascular malformations, trauma-related proliferation, and hormonal influences [9]. Because IMH mimics common musculoskeletal pathology, diagnosis is frequently delayed, particularly in active young patients who present with chronic upper- or lower-extremity pain [10]. This case highlights the importance of including IMH in the differential diagnosis for longstanding periarticular pain and illustrates key considerations in diagnostic evaluation and management.

IMHs are thought to arise from aberrant vascular proliferation within skeletal muscle, with gradual enlargement over time. Plausible mechanisms include congenital vascular malformations that may become symptomatic following trauma or increased mechanical stress [11]. In athletes, microtrauma and repetitive loading may exacerbate pain through intralesional hemorrhage, inflammation, or venous congestion [12]. These lesions are difficult to detect on physical examination because of their deep intramuscular location, which contributes to diagnostic delay and to misattribution of symptoms to more common orthopedic conditions.

Current literature describes IMHs as a commonly missed diagnosis because they often present similarly to other knee pathologies [2,9,13]. Reviews most often describe IMHs in the thigh and calf [2,7]. Chronic pain, often exercise-related, may be the most common symptom rather than a prominent palpable mass [13,14], and clinical suspicion is difficult to establish without imaging [11].

In this case, a 17-year-old student athlete presented with chronic anteromedial knee pain and a small periarticular intramuscular mass near the vastus medialis insertion, with MRI interpreted as most consistent with a ganglion cyst. This mirrors existing literature on pediatric and adolescent cases [13] in which periarticular intramuscular vascular lesions of the vastus medialis and vastus intermedius region, treated with surgical excision and confirmed by histopathology, demonstrated no evidence of recurrence.

Imaging literature further emphasizes that IMHs can contain variable proportions of vascular spaces, fibrous septa, smooth muscle, and blood products [8]. This heterogeneity produces MRI images with areas of variable enhancement or internal complexity, which may be mistaken for simple cystic lesions when the vascular nature is not appreciated [15]. On histopathology, our patient’s lesion demonstrated dilated, blood-filled vascular channels lined by flattened endothelium and dissecting between skeletal muscle fibers, consistent with the cavernous subtype originally described by Allen and Enzinger [2]. The presence of intratumoral skeletal muscle entrapment, a defining feature of IMH, helps distinguish IMH from a true vascular malformation occupying the same anatomic plane [2,6,7]. Reported local recurrence rates after surgical excision range widely and correlate roughly with margin status. Bella et al. demonstrated that microscopically positive margins significantly increase recurrence risk, supporting wide local excision when functionally feasible [7,14,16]. In the present case, gross resection with a circumferential cuff of normal-appearing muscle was performed. Brown and colleagues described a comparable diagnostic delay in a competitive basketball player whose IMH of the thigh had been treated for more than a year as recurrent muscle strain before MRI prompted a definitive diagnosis. This underscores the athletic context masking the underlying lesion [14]. Given the patient’s persistent, activity-limiting symptoms and an intramuscular cystic-appearing lesion on MRI, surgical excision served as both the diagnostic and therapeutic approach, given the low suspicion for malignancy. The patient also elected against continued nonoperative management because of persistent symptoms over several months with ongoing limitation in sports participation. Biopsy with referral to orthopedic oncology was discussed, but the patient preferred to pursue management locally. Surgical pathology confirmed IMH following excision. Overall, this case reinforces prior literature suggesting that periarticular IMHs are an easily missed diagnosis in adolescent athletes and should be considered when focal, chronic pain coexists with a deep soft-tissue lesion.

## Conclusions

This case highlights IMH as a rare but important cause of chronic anteromedial knee pain in an adolescent athlete, particularly when imaging suggests a benign cystic process. Although preoperative evaluation pointed to a ganglion cyst as the leading preoperative diagnosis, histopathology confirmed an IMH following excision, and short-term follow-up demonstrated improved pain without acute complications.

Clinicians should consider intramuscular vascular tumors in the differential diagnosis of persistent periarticular pain accompanied by a deep soft-tissue lesion, to reduce diagnostic delay and guide timely surgical planning. Heightened awareness in the evaluation of young, active patients may help avoid unsuccessful nonoperative trials, guide appropriate referral, and preserve function through definitive surgical excision.
